# Burden of disease attributable to Risk Factors in Brazil: an analysis of national and subnational estimates from the 2019 Global Burden of Disease study

**DOI:** 10.1590/0037-8682-0262-2021

**Published:** 2022-01-28

**Authors:** Deborah Carvalho Malta, Mariana Santos Felisbino-Mendes, Ísis Eloah Machado, Guilherme Augusto Veloso, Crizian Saar Gomes, Luisa Campos Caldeira Brant, Antonio Luiz Pinho Ribeiro, Patrícia Pereira Vasconcelos de Oliveira, Luisa Sorio Flor, Emmanuela Gakidou

**Affiliations:** 1 Universidade Federal de Minas Gerais, Escola de Enfermagem, Departamento de Enfermagem Materno-Infantil e Saúde Pública, Belo Horizonte, MG, Brasil.; 2 Universidade Federal de Ouro Preto, Escola de Medicina, Departamento de Medicina da Família, Saúde Mental e Coletiva, Ouro Preto, MG, Brasil.; 3 Universidade Federal de Minas Gerais, Departamento de Estatística, Belo Horizonte, MG, Brazil.; 4 Universidade Federal de Minas Gerais, Faculdade de Medicina, Departamento de Medicina Preventiva e Social, Programa de Pós-Graduação em Saúde Pública, Belo Horizonte, MG, Brasil.; 5 Universidade Federal de Minas Gerais, Faculdade de Medicina e Hospital das Clínicas, Departamento de Clínica Médica, Belo Horizonte, MG, Brasil.; 6 Ministério da Saúde, Secretaria de Vigilância em Saúde, Brasília, DF, Brasil.; 7 University of Washington, Institute for Health Metrics and Evaluation, Seattle, Washington, United States of America.

**Keywords:** Risk factors, Mortality premature, Disability-adjusted life years, Global Burden of Disease, Brazil

## Abstract

**INTRODUCTION:**

Monitoring trends in risk factors (RFs) and the burden of diseases attributable to exposure to RFs is an important measure to identify public health advances and current inadequate efforts. Objective: Analyze the global burden of disease attributable to exposure RFs in Brazil, and its changes from 1990 to 2019, according to the sex and age group.

**METHODS:**

This study used data from the Global Burden of Disease study. The Summary Exposure Value, which represents weighted prevalence by risk, was used to estimate exposure to RFs. The mortality and DALYs (Disability Adjusted Life Years) measurements were used to estimate the burden of diseases. For comparisons by year and between Brazilian states, age-standardized rates were used.

**RESULTS:**

Arterial hypertension was the factor responsible for most deaths in both sexes. For DALYs, the most important RF was the high body mass index (BMI) for women and alcohol consumption for men. Smoking had a substantial reduction in the attributable burden of deaths in the period. An important reduction was identified in the exposure to RFs related to socioeconomic development, such as unsafe water, lack of sanitation, and child malnutrition. Metabolic RFs, such as high BMI, hypertension, and alcohol consumption showed an increase in the attributable burden.

**CONCLUSIONS:**

Our findings point to an increase in metabolic RFs, which are the main RFs for mortality and DALYs. These results can help to consolidate and strengthen public policies that promote healthy lifestyles, thus reducing disease and death.

## INTRODUCTION

The monitoring of the occurrence of risk factors (RFs), as well as of the burden of diseases attributable to exposure to RFs, contributes to the understanding of those factors’ dynamics and their variation over time, allowing for the evaluation of the effectiveness of public policies, their progresses, failures, and challenges, thus making improvements in those public health policies possible. The term ‘Risk Factor’ means “some type of exposure that causes a loss of health in the population”^1^.

The RFs that interfere in the health-disease process include non-modifiable factors (genetic heritage, age, sex, race/ethnicity); proximal factors that are associated with specific behaviors and can be modified (smoking, physical inactivity, and inadequate eating); intermediate factors, such as metabolic risks; occupational factors and environmental exposure; and more distal factors that are hierarchical and determine the others. The change in the last set of RFs is determined by structural interventions (income, education, poverty) and, in most cases by implementing inter-sector policies^2^- ^5^.

The Global Burden of Disease (GBD) study estimates the population burden attributable to RFs using standardized methods for a wide set of RFs and encompassing all countries. A pre-analysis of the burden attributable to the group of RF of the GDB was conducted with estimates from the 2015 GBD^3^ and later, in 2016, with revised estimates^6^, showing that a unhealthy diet, metabolic RFs, alcohol, and tobacco consumption are important protagonists in the overall death and disease burden in Brazil. More recent studies evaluated the burden attributable to specific RFs, such as hypertension^7^, obesity^8^, hyperglycemia^9^, physical inactivity^10^, and smoking^11^. However, those estimates were not calculated together, which hindered the comparison between the RFs and their consequences in the overall disease burden. 

In 2019, the GBD estimates showed new advancements, especially in the extensive revisions of Risk-Outcome pairs, including methodology innovation related to the evaluation of risk exposure, as well as the updating of the data on exposure and related risks, and the inclusion of two new RFs. Therefore, it is important to update the RF estimates and make advances in the knowledge on the loss of health and the disease burdens attributable to these factors. 

The present study sought to analyze the overall disease burden attributable to RFs in Brazil, and its changes from 1990 to 2019, according to sex and age groups. 

## METHODS

This study analyzed estimates of RFs in Brazil, acquired from the 2019 GBD study databank, from the Institute of Health Metrics and Evaluation (IHME), available at http://ghdx.healthdata.org/.

The GBD uses a hierarchical list of RFs, analyzed at four levels. Level 1 separates these risks in three overarching groups: metabolic, behavioral, and environmental. Level 2 details the RF in smaller groups and includes 20 single RFs or risk clusters. Level 3 includes disaggregated single risks from within Level 2 risk clusters, comprising 52 RFs or RF clusters, and Level 4 includes 69 specific RFs. Combined, in 2019, the GBD study analyzed 87 risk factors. Detailed information on RFs by Level is available elsewhere^12^. The present study used hierarchical Levels 1, 2, and 4.

To calculate the burden attributable to RFs, the GBD followed the structure established for comparative risk assessment (CRA). The CRA has 5 main steps: 

### 1) To estimate the level of exposure through available sources

The first step consists of the identification of the available data sources through the search and identification of data related to each RF. There are five components in the identification of the data sources: Global Health Data Exchange (GHDx), which gathers the published data; systematic literature reviews; access to the data made available by GBD collaborators; guided research on the internet; and other data. The GBD data catalog has more than 75,000 records, including residential surveys, administrative data, census, vital records, environmental measurements, as well as data about commerce, sales, and consumption^12^. More than 200 sources of data were consulted for Brazil, including the National Health Survey (PNS, in Portuguese), Surveillance for risk factors and protection against chronic diseases by telephone inquiry (VIGITEL), the National Household Survey Sample (PNAD, in Portuguese), the National School-based Student Health Survey (PeNSE, in Portuguese), among others^3^.

Specific methods to estimate mean levels of exposure by age-sex-location-year varied across RFs and are described elsewhere^12^. In general, adjustments were made to the data to correct for bias in self-reported information and under-reporting, as well as to adjust alternative definitions to the same standardized reference definition. Adjustments for both sex and standardized age groups were also applied when needed. These adjustments are sometimes necessary, since not every data source uses the same exposure definition, survey questions, methods, or report results for the same population groups^12^. For this purpose, a statistical relationship between the reference and alternative methods of ascertainment was estimated through a network meta-regression and used to correct the alternative data using this relationship. Finally, exposure data were mainly modelled using either spatiotemporal Gaussian process regression (ST-GPR) or DisMod-MR 2.1, which are Bayesian statistical models that pool data from different sources and generate final exposure estimates with uncertainty for each year, location, sex, and age group^13^.

### 2) To estimate pairs of risk factors and their outcomes

In this phase, risk-outcome pairs included in this study were identified according to the available literature. Literature review and meta-analysis were performed for each risk to identify risk-outcome pairs, for which convincing evidence to support a causal relationship was available. In the GBD 2019, 47 new risk-outcome pairs were added and 12 were excluded based on the statistical association found in the review. In the end, 560 risk-outcome pairs were included in the analysis^12^.

### 3) Estimating relative risks as a function of exposure

Relative risks (RR) by level of exposure or by cause of mortality or morbidity are identified in published systematic revisions, and IHME updates these where necessary to include any new studies that become available. In 2019, 81 new systematic reviews were performed. The new data allowed for the inclusion of two new RFs (high and low temperature)^12^. The available evidence is summarized through meta-analysis and meta-regression methods. The RR used may be in continuous or categorical format, depending on availability in the literature. For continuous risks, risk outcome pairs were estimated by assuming non-linear or log-linear relationships. Mathematical and computational details, as well as risk-specific approaches, are described in a different publication^12^. 

### 4) Estimating the Theoretical Minimum Risk Exposure Level (TMREL)

The TMREL is defined as the minimum level of exposure for each RF, in which the event occurrence is the least likely. The aim is to measure how much the disease burden would be reduced if, in the past, the exposure of the population had been modified to a theoretical minimum level of exposure risk^3^ ^,^ ^12^. Studies and meta-analyses are used to establish the minimum level of exposure for each risk factor. For instance, the TMREL, or minimum risk exposure level, would be: for fruits and vegetables, a daily consumption of 200 to 400 g; for physical activity, 8,000 metabolic equivalent of task (METs) per day; for BMI, between 21 and 23 kg/m^2^; for sodium intake, 1 to 5 g, among others^4^ ^,^ ^12^. For those risks with a dichotomous exposure, the TMREL is the absence of exposure. 

### Estimating the population attributable fraction

The population attributable fraction (PAF) is the proportion of risk that would be reduced in a given year if the exposure to a risk in the past was reduced to an ideal exposure scenario^1^. PAF was calculated for each risk by age-sex-location-year, using the formulas described in the Supplementary Material. 

The GBD also uses the concept known as Summary Exposure Value (SEV). The SEV shows the prevalence weighted by the risk. The SEV scale ranges from 0% to 100%, considering that 0% represents no exposure to risk and 100% represents maximum exposure. The decline in SEV indicates reduced exposure, while an increase in SEV represents the opposite. Further detail concerning the SEV is also available elsewhere^3^ ^,^ ^4^.

In the current study, the burden attributable to different RFs was estimated according to sex. The measures of burden used in this analysis included: death, mortality rates, and disability-adjusted life years (DALYs). The 95% uncertainty interval (95% UI) was also reported. A ranking of the RFs was created to highlight changes that occurred between 1990 and 2019, according to sex and age group, as well as a ranking of the RFs for each of the 27 states of Brazil in 2019. For the comparisons between states over time, age standardized rates were used. For the other analyses, non-standardized rates were shown. 

Finally, the selected RFs those that changed the most between 1990 and 2019 were analyzed according to the Socio-Demographic Index (SDI), which is a composite index measuring per capita income, fertility, and education. The index ranges from 0 (least developed) to 1 (most developed) and enables the comparison between different geographic realities according to their development. 

The analysis was conducted in an RStudio (RStudio Team, 2019), and figures were produced using the ggplot2 package^14^.

The GBD-Brazil project was approved by the Research Ethics Committee from the Federal University of Minas Gerais (UFMG), logged under CAAE Project number - 62803316.7.0000.5149.

## RESULTS

Table 1 shows the age standardized SEV for selected risks in Brazil in 1990 and 2019. Between those years, the largest SEV reductions for both sexes combined occurred for air pollution (-60.0%); other environmental risks (-38.0%); tobacco use (-34.0%); unsafe water, sanitation, and handwashing (-28.0%); and child and maternal malnutrition (-18.0%). Sex differences were found in the reduction of tobacco use, with larger reductions among males when compared to females. For some risks with high SEV, a small change was identified between 1990 and 2019, particularly for dietary risks and low physical activity. Increases in SEV were observed for high BMI (+110%), alcohol consumption (+41%), drug use (+20%), high fasting plasma glucose (+15%), high LDL-cholesterol (+12%), and kidney dysfunction (+12%). For high systolic blood pressure, the variation was close to zero for both sexes combined. However, there was a reduction of 8% for women and an increase of 10% for men. 

**TABLE 1:** Age-standardized synthesis of risk exposure (SEV) for selected RFs (Level 2), according to sex, in 1990 and 2019, and percentage (%) of change in the period, GBD Brazil, 2019.

Risk Factor	Both			Female			Male		
	1990	2019	PC (%)	1990	2019	PC (%)	1990	2019	PC (%)
**All risk factors**	20.9	20.9	0	19.4	19.7	2	22.6	22.2	-2
	(17.4;24.5)	(17.1;24.9)	(-6;5)	(16.8;22.4)	(16.8;22.9)	(-4;8)	(17.8;27.1)	(17;27.3)	(-9;6)
Air pollution*	23.3	9.3	-60	23.3	9.3	-60	23.4	9.4	-60
	(14.7;33.9)	(5.9;13.3)	(-40;-74)	(14.7;33.7)	(5.8;13.4)	(-74;-40)	(14.4;33.9)	(5.9;13.4)	(-73;-40)
**Alcohol use**	6.6	9.3	41	3.3	4.9	49	10	13.8	38
	(4.6;9)	(6.4;12.5)	(24;63)	(2.1;4.9)	(3.2;7.1)	(22;83)	(7.1;13.5)	(9.9;18.4)	(21;61)
**Child and maternal malnutrition**	20.3	16.7	-18	20.3	16.7	-18	0.9	0.8	-18
	(18.1;22.7)	(14.7;18.6)	(-7;-29)	(18.1;22.7)	(14.7;18.6)	(-29;-7)	(0.8;1.1)	(0.6;0.9)	(-26;-11)
**Childhood sexual abuse and bullying**	5.3	5.8	9	5.6	6.1	9	5	5.5	9
	(3.6;7.8)	(3.8;8.9)	(-2;21)	(3.6;8.2)	(3.9;9.2)	(-3;22)	(3.1;7.9)	(3.4;8.9)	(-5;24)
**Dietary risks**	39.6	39.3	-1	36	35.6	-1	43.5	43.3	-1
	(24.7;55.2)	(25.4;53.7)	(-12;14)	(23.2;52.2)	(24.6;50.1)	(-13;13)	(25;59.6)	(25.4;59.2)	(-17;20)
**Drug use**	0.2	0.3	20	0.2	0.2	13	0.3	0.3	25
	(0.1;0.4)	(0.1;0.5)	(9;34)	(0.1;0.3)	(0.1;0.4)	(5;24)	(0.1;0.5)	(0.2;0.6)	(10;43)
High body-mass index*	14.9	31.2	110	16.2	31.9	97	13.4	30.4	127
	(10.4;20.5)	(25.5;39.5)	(79;162)	(11.4;22.7)	(26;40)	(65;146)	(9.1;19.5)	(24;38.9)	(88;197)
**High fasting plasma glucose**	9.9	11.4	15	9.6	10.7	12	10.3	12.3	19
	(8.7;11.1)	(10.1;12.8)	(9;21)	(8.4;10.8)	(9.5;12)	(4;20)	(9;11.6)	(10.8;13.9)	(11;27)
**High LDL cholesterol**	40.3	45.1	12	41.2	46	12	39.3	43.9	12
	(37;43.7)	(41.9;48.3)	(7;17)	(37.8;44.7)	(42.6;49.4)	(6;19)	(35.8;42.9)	(40.3;47.6)	(5;20)
**High systolic blood pressure**	27.1	27.3	1	26.1	24	-8	28.1	30.8	10
	(24.8;29.6)	(25.2;29.3)	(-7;8)	(23.1;29.4)	(21.7;26.3)	(-19;4)	(25.4;30.6)	(28.4;33.2)	(1;19)
**Intimate partner violence**	12.4	12.6	2	12.4	12.6	2			
	(7;19.3)	(7.2;19.5)	(1;4)	(7;19.3)	(7.2;19.5)	(1;4)			
**Kidney dysfunction**	18.9	21.2	12	19	21.4	12	18.7	20.9	12
	(13;26.2)	(15.1;28.6)	(8;17)	(13.1;26.4)	(15.2;29)	(9;18)	(12.8;25.9)	(14.8;28.2)	(8;18)
**Low bone mineral density**	17.3	15.5	-10	22.4	20.3	-9	11.6	9.9	-14
	(12.1;24)	(10.3;22.1)	(-5;-16)	(16.4;29.6)	(14;27.6)	(-16;-3)	(7;18.2)	(5.6;15.9)	(-24;-7)
**Low physical activity**	12	12.5	4	12.6	13.2	5	11.4	11.8	3
	(7.2;19.5)	(7.7;19.2)	(-5;18)	(8;19.1)	(8.6;19.2)	(-3;18)	(6.3;19.5)	(6.7;19.9)	(-8;20)
**Non-optimal temperature**	24	23.2	-4	24	23.2	-4	24	23.2	-4
	(18.1;31.3)	(17.5;30.6)	(1;-8)	(18.1;31.3)	(17.5;30.6)	(-8;1)	(18.2;31.3)	(17.5;30.6)	(-8;1)
**Occupational risks**	3.2	3.4	6	2.2	2.7	22	4.1	4	-3
	(2.8;3.7)	(3;3.9)	(-1;15)	(1.8;2.8)	(2.4;3.2)	(9;39)	(3.5;4.8)	(3.5;4.7)	(-11;7)
**Other environmental risks**	50.9	31.4	-38	45.4	27.8	-39	56.7	35.4	-38
	(36.9;61.8)	(19.7;44.7)	(-27;-48)	(29.2;57.9)	(15.9;41.6)	(-48;-28)	(45;66)	(23.9;48.4)	(-47;-26)
Tobacco*	33.2	22	-34	30.2	20.7	-31	36.4	23.5	-36
	(31.1;35.4)	(20.4;23.7)	(-29;-38)	(28;32.5)	(18.9;22.6)	(-37;-25)	(34.1;38.9)	(21.1;25.9)	(-42;-29)
Unsafe water, sanitation, and handwashing*	55.2	39.5	-28	69.3	60.4	-13	40.5	17.7	-56
	(53.6;56.7)	(38.5;40.7)	(-26;-31)	(68.7;70)	(59.5;61)	(-14;-12)	(37.6;43.3)	(16;19.8)	(-61;-51)

*Risk factors whose uncertainty intervals in 1990 and 2019 do not overlap, for both, female and male. **PC:** Percentage Change.

Supplementary Material (Figure S1A and Figure S1B) shows the mortality burden attributable to Level 1 RFs in 1990 and 2019. In 2019, 60.5% of the deaths by non-communicable diseases (NCD) are due to RFs. On the other hand, only 50% of infectious maternal causes and 24.1% of the external causes were attributable to RFs. All RFs combined were responsible for 60.6% and 54.8% of the deaths in 1990 and 2019, respectively. 

**FIGURE 1:**
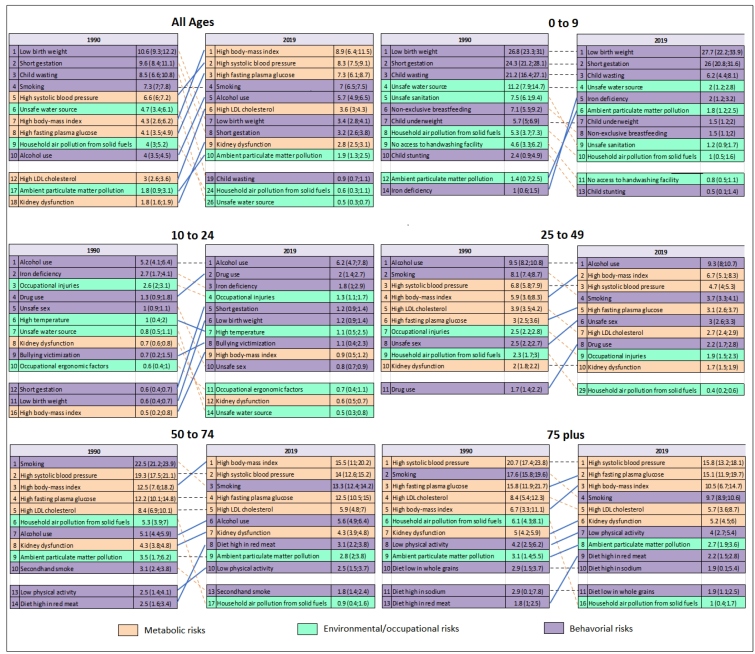
Changes in the ranking of percentage of DALYs attributable to RFs (Level 4) by age groups in 1990 and 2019, GBD Brazil, 2019**.**

The Figure 1 presents the ranking of percentage of DALYs attributable to level 4 RFs for both sexes combined by age groups in 1990 and 2019. For all ages, a significant decrease was observed in low birth weight, short gestation, and environmental RFs (unsafe water source, household air pollution from solid fuels) between 1990 and 2019. During this period, high BMI, high systolic blood pressure, and high fasting plasma glucose moved up to the top three positions in the ranking, followed by smoking (4^th^), alcohol consumption (5^th^), and high LDL cholesterol (6^th^). In terms of age groups, in 2019, the most important RF for the 0 to 9 age group were low birth weight, short gestation, and child wasting. For 10 to 24 years of age, alcohol consumption, drug use, and iron deficiency reached the top rankings. For the age group of 25 to 49 years, the most prominent risks were alcohol use, high BMI, and high systolic blood pressure. For the age group of 50 to 74 years, high BMI, high systolic blood pressure and smoking were the top three. Finally, for the age group of 75 years and above, high systolic blood pressure, high fasting plasma glucose, and high BMI were the leading risk factors (Figure 1).

**FIGURE 2:**
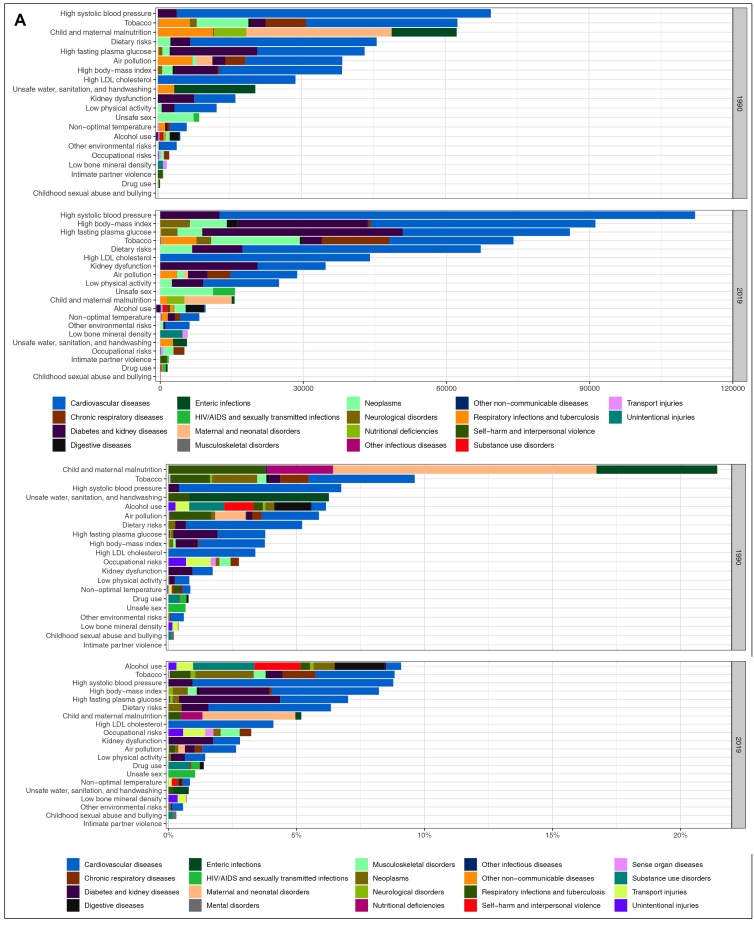
Number of deaths attributable to risk factors (Level 2) for women (A) and for men (B) for all age groups. GBD Brazil, 2019.

Figure 2 shows the attributable deaths by causes for females (A) and males (B) for Level 2 RFs in 1990 and 2019. In 2019, high systolic blood pressure was the leading RF for mortality in Brazil, particularly due to, cardiovascular conditions, contributing to more than 239,000 deaths, approximately 127,000 of those among males and 112,000 among females. Tobacco, the second most important RF for males and fourth for females, contributed to approximately 190,500 deaths due to cardiovascular diseases, neoplasms, chronic respiratory diseases, and other conditions across the sexes in 2019. High BMI, fasting plasma glucose, and inadequate diet also contributed largely to cardiovascular diseases, diabetes, and kidney diseases deaths for both sexes. Supplementary Material (Figures S2 A and B) show that high BMI and high blood pressure are the main RFs contributing to DALYs for women in 2019, whereas for men, the main RFs are alcohol use and tobacco. 

**FIGURE 3:**
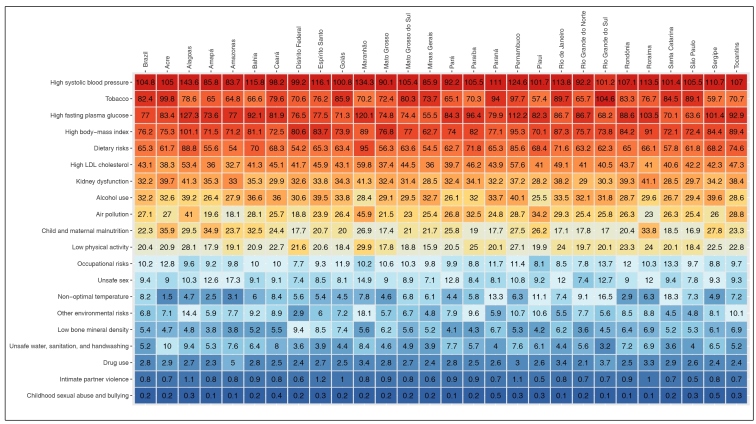
Ranking of RFs (Level 2) according to all-cause age-standardized mortality rates for Brazil and its states in 2019, GBD Brazil, 2019.

Figure 3 shows the ranking of all-cause age-standardized mortality rate (per 100,000) attributable to the selected Level 2 RF for both sexes for Brazil and its states in 2019. Each column has 20 shades, ranging from darker red tones (higher death rates) to darker blue tones (lower death rates). High systolic blood pressure was ranked number one in every state except Rio Grande do Sul, where tobacco ranked number one. Tobacco, high fasting plasma glucose, high BMI, and dietary risks were among the five main RFs in the vast majority of the Brazilian states. Maternal and child malnutrition ranked 8^th^ in Acre, Amapá, and Roraima, and 9^th^ in Bahia, Amazonas, and Piaui. Supplementary Material (Figure S3) shows the ranking of age-standardized DALY rates (per 100,000) attributable to the same selected Level 2 RF. 

**FIGURE 4:**
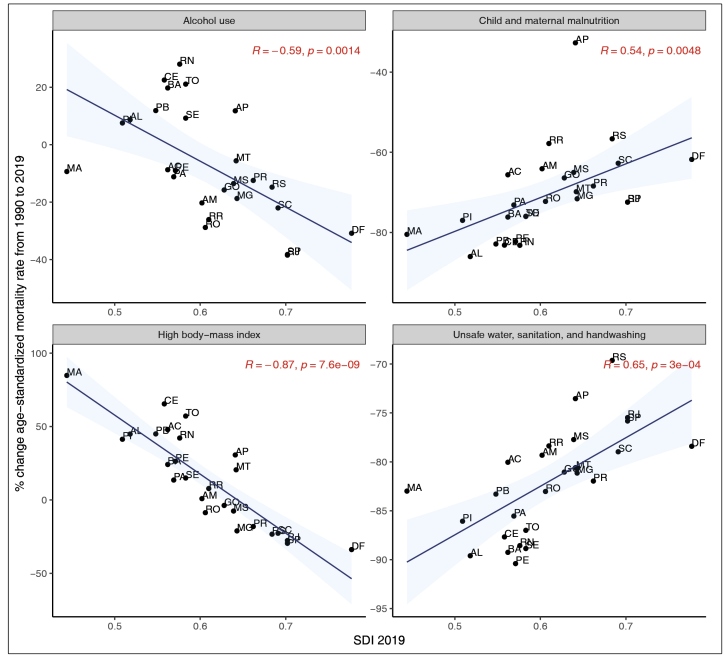
Socio-Demographic Index and changes in mortality rates attributable to selected RFs (alcohol, high BMI, unsafe water and sanitation, and child and maternal malnutrition) between 1990 and 2019, by state.

Figure 4 shows the relative change in mortality rates attributable to selected RF according to SDI levels, along with the correlation coefficient and the p-value associated with it. Between 1990 and 2019, an increase in death rates attributable to high BMI and alcohol consumption was identified in some of the states with the lowest SDI, (Rio Grande do Norte (RN), Ceará (CE), Paraíba (PB), Alagoas (AL)), and a reduction in the states with the highest SDI (Federal District (DF), Rio de Janeiro, São Paulo (SP), Santa Catarina (SC)). Deaths attributable to sanitation risks showed a decrease in every state; however, the most significant reduction occurred in those states with the lowest SDI values (Alagoas (AL), Bahia (BA), Pernambuco (PE)), as compared to those with the highest SDIs (Rio Grande do Sul (RS), Rio de Janeiro (RJ), São Paulo (SP)). Maternal and child malnutrition mortality rates also showed a reduction in every state, with a more expressive reduction found in the states with the lowest SDIs (Maranhão (MA), Alagoas (AL)). Amapá (AP), which has a low SDI, presented the least reduction in malnutrition (-30%). For further details, see Supplementary Material (Table S1) with detailed information from each state.

## DISCUSSION

The burden of diseases attributable to RFs in Brazil varied considerably in 2019 between men and women, age groups, and states. Metabolic factors, such as high blood pressure, were the leading RFs for mortality for both men and women in the country. In terms of DALYs, the most prominent RFs were high BMI for women and alcohol consumption for men. Tobacco remains one of the most important RFs for both mortality and burden, especially for males. Analysis by state shows that high BMI and high blood pressure are important RFs for mortality and DALYs throughout Brazil. Child and maternal malnutrition, however, accounted for the highest DALY rate in ten states, while tobacco was the leading RF in Rio Grande do Sul and São Paulo. 

These findings, in general, follow global and national trends^7^ ^,^ ^12^. In GBD 2019, an important change in terms of dietary risks was observed in the ranking, as compared to the estimates from GBD 2015. This may well be a result of data availability and methodological advances incorporated into the current GBD study, which resulted in the exclusion of some dietary risk-outcome pairs, as they did not meet the inclusion criteria^12^. It should also be emphasized that, regardless of this update, dietary risks continue to be one of the top five risk factors for mortality in the ranking, for Brazil and for the majority of its states. A greater importance of the metabolic risks was also noted, which corroborates studies about the Brazilian population based on laboratory data. Those studies indicate that at least one in every three Brazilians has the metabolic syndrome phenotype, especially in women^8^. By contrast, it should also be highlighted that a large portion of the metabolic RFs are intermediate risk factors in the chain of causality of the NCDs, which can be considered aggravating factors and have primary factors as their cause. Therefore, the public policies that promote health and primary preventive actions continue to be essential in modifying these behaviors (healthy diet, physical activity, no drinking, no smoking). The establishment of integrated actions among the various segments and areas of society, such as governments, non-governmental organizations, universities, can be an important strategy to protect future generations and coordinate the response over the country. These actions would contribute to the reduction in intermediate RFs and, consequently, to the reduction of the global burden of diseases^3^ ^,^ ^15^ ^,^ ^16^. One example of the importance of public policies of health promotion were the actions against tobacco use, which resulted in its reduction throughout the studied period due to new regulations^11^. 

This study reveals major regional inequalities. When comparing SDIs, the wealthier states presented better results overall. Deaths attributable to high BMI have increased in states with low SDIs and have reduced in states with high SDIs. By contrast, sharper and more expressive declines in factors related to sanitation and malnutrition occurred in states with low SDIs, a reflection of recent efforts to improve sanitation and primary care^6^. These RFs (unsafe water, lack of sanitation, not washing hands, infant malnutrition) are more heavily connected to socioeconomic development, and the reduction in these factors contributes significantly to the decrease in infant mortality^17^.

However, even with a significant reduction of the burden attributable to maternal and child malnutrition, this risk factor continues to be important in some age groups and in some states (mainly states of the Northeast and North regions). In ten states, it ranked first as the most important RFs contributing to DALYs. To improve this situation, specific measures are recommended, such as providing more services to this segment of the population, including better antenatal and reproductive care, reduction in the inequalities caused by economic growth and urbanization, and better education. The Brazilian “Bolsa Família” cash transfer program, which has contributed to improving social and health outcomes among its beneficiaries, might also need to consider these regional differences^18^. These findings may also indicate setbacks caused by the interruption of policies due to reductions in funding and social programs, as well as the limitation of investments in public health^19^.

It can therefore be concluded that this study is beneficial, since the estimates made in 2019 allow us to evaluate the burden of diseases attributable to risk factors. It is notable that Brazil plays an important role in GBD estimates, as can be seen in the country's ability to produce studies and surveys on risk factors with verifiable quality^20^ ^,^ ^22^, including the National Health Survey^23^, surveys by telephone^24^, the National School-based Student Health Survey^21^, laboratory data^25^, surveillance at emergency entry points^26^, and longitudinal follow-up studies^27^ ^,^ ^28^.

As regards limitations, GBD assumes that most relative risks are distributed in a uniform manner in all countries, for a given age and sex, which must be evaluated cautiously, since variations may well exist between countries. Moreover, not all RFs were included in this study, since there are not enough studies on all risks, especially those referring to social determinants of health, as some risks can use unreliable data^3^ ^,^ ^4^.

Our findings point to the ranking of exposure of the Brazilian population to selected RFs, and the quantification of the impact of that exposure on health, highlighting metabolic and behavioral risks. This evidence can help to consolidate and strengthen public policies that promote healthy lifestyles and aid in reducing risk factors, thus contributing to the improvement of the health of the Brazilian population and reducing disease and deaths.
