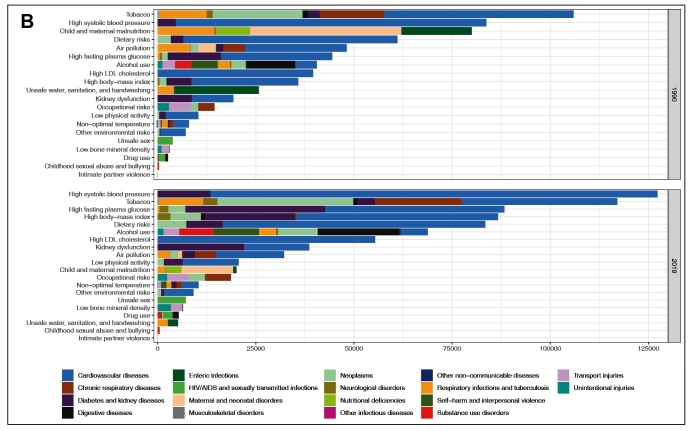# Erratum

**DOI:** 10.1590/0037-8682-0262B-2021

**Published:** 2022-02-25

Revista da Sociedade Brasileira de Medicina Tropical/Journal of the Brazilian Society of Tropical Medicine

**Title:** Burden of disease attributable to Risk Factors in Brazil: an analysis of national and subnational estimates from the 2019 Global Burden of Disease study 

**Vol.:55(suppl 1): 2022 - Page: 6/10** - doi: 10.1590/0037-8682-0262-2021 - **FIGURE 2B**

**Figure:**
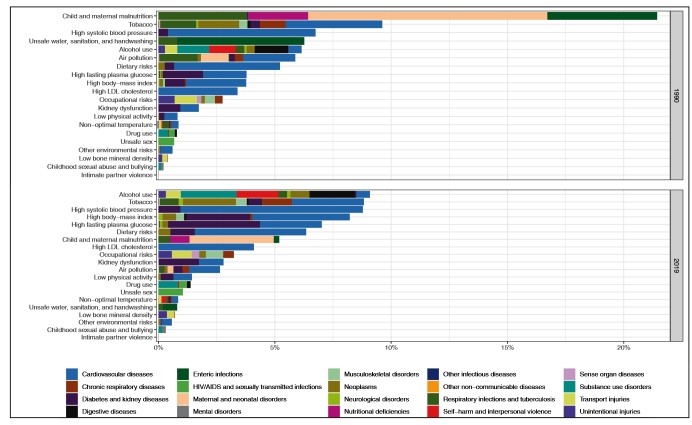

**Should read:**

**Figure:**