# Supplementary material for: Burden of disease attributable to Risk Factors in Brazil: an analysis of national and subnational estimates from the 2019 Global Burden of Disease study
Source: Rev Soc Bras Med Trop. 2022 Jan 28;55(Suppl 1):e0262-2021. doi: 10.1590/0037-8682-0262-2021 (PMC9009437; doi:10.1590/0037-8682-0262-2021)
Supplement: Supplementary file 1 [file 1678-9849-rsbmt-55-s01-e0262-2021-supp1.pdf]

The PAF is a function of exposure (prevalence – P), RR, TMREL, and is defined for risks with a continuous exposure as:

$$PAF_{joasgt} = \frac{\int_{x=l}^{u} RR_{joasg}(x)P_{jasgt}(x)dx - RR_{joasg}(TMREL_{jas})}{\int_{x=l}^{u} RR_{joasg}(x)P_{jasgt}(x)dx}$$

Where $PAF_{joasgt}$ is the PAF for cause *o*, for age group *a*, sex *s*, location *g*, and year *t*; $RR_{joasg}(x)$ is the relative risk as a function of exposure level *x* for risk factor *j*, for cause *o* controlled for confounding, age group *a*, sex *s*, and location *g*, with the lowest level of observed exposure as *l* and the highest as *u*; $P_{jasgt}(x)$ is the distribution of exposure at *x* for age group *a*, sex *s*, location *g*, and year *t*; and $TMREL_{jas}$ is the TMREL for RF *j*, age group *a*, and sex *s*.

Where risk exposure is dichotomous or polytomous, this formula is simplified to the discrete form of the equation:

$$PAF = \frac{\sum_{x=0}^{1} RR(x)P(x) - RR(TMREL)}{\sum_{x=0}^{1} RR(x)P(x)}$$

To estimate the burden attributable to a given risk factor, each year-location-sex-age specific PAF is multiplied by the year-location-sex-age metric of burden (e.g. number of deaths) for the desired specific cause relevant to the risk. For the combined effects of RFs, a mediation matrix was developed by IHME to try to correct for overestimation of the PAF and the attributable burden for combinations of risks if we were to simply assume independence with no mediation[12].
