# Supplementary material for: Burden of disease attributable to Risk Factors in Brazil: an analysis of national and subnational estimates from the 2019 Global Burden of Disease study
Source: Rev Soc Bras Med Trop. 2022 Jan 28;55(Suppl 1):e0262-2021. doi: 10.1590/0037-8682-0262-2021 (PMC9009437; doi:10.1590/0037-8682-0262-2021)
Supplement: Supplementary file 2 [file 1678-9849-rsbmt-55-s01-e0262-2021-supp2.pdf]

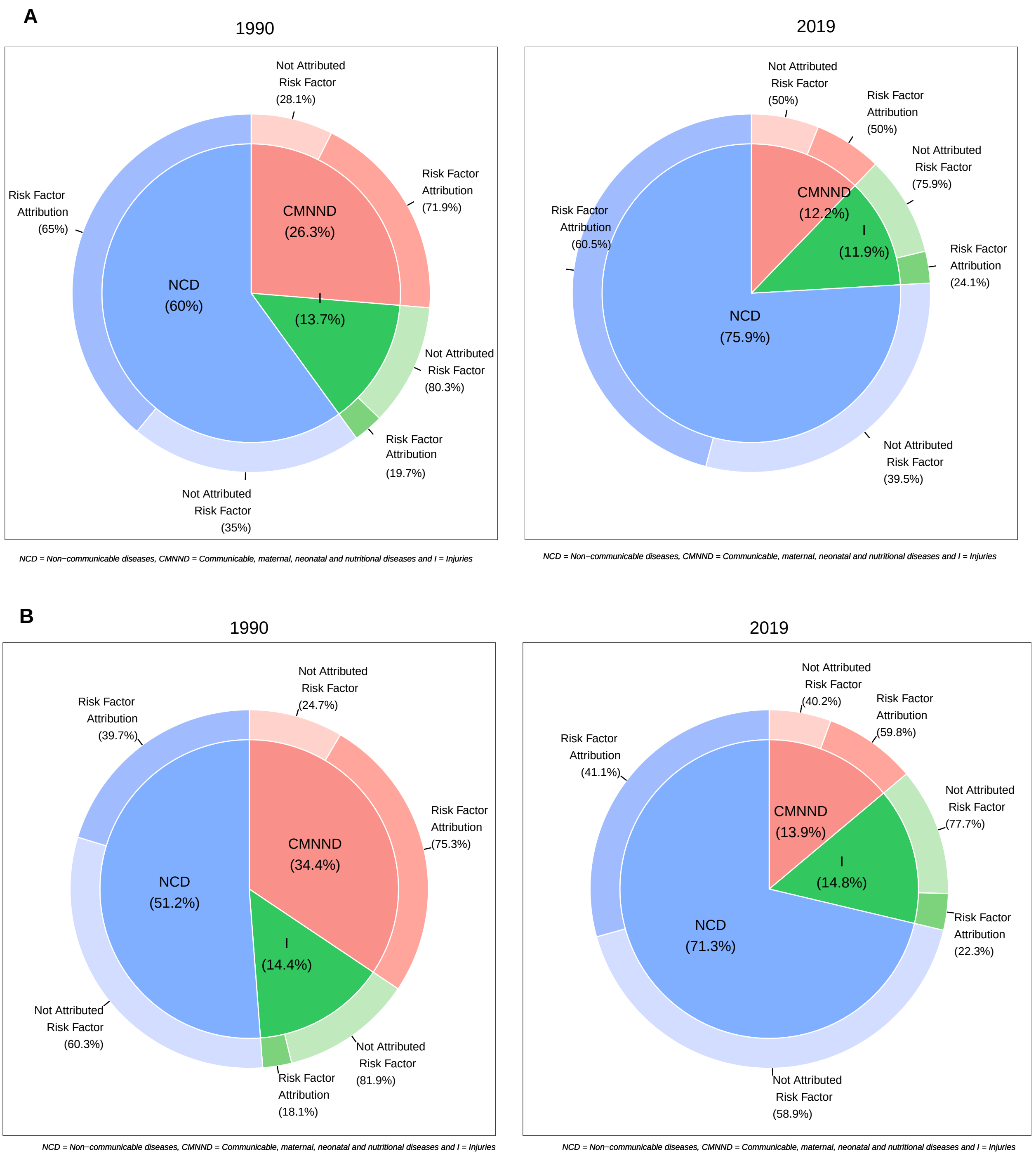

SUPPLEMENTARY FIGURE 1: Burden of diseases attributable to Level 1 RFs for both sexes considering deaths (A) and DALYS (B) in 1990 and 2019, all ages. GBD Brazil, 2019.

In 1990, 60% of all deaths were caused by NCDs, with 65% attributable to risk factors (Figure S1A). In 2019, 75.9% of the deaths were caused by NCDs and 60.4% were attributed to risk factors. There was also a relevant reduction in the burden of mortality attributable to risk factors, by communicable, maternal, and nutritional-neonatal causes, from 71.9% in 1990 to 50.0% in 2019. This reveals the progress in the reduction of deaths by avoidable causes in this group. In the case of deaths by external causes, there was an increase in the burden attributable to risk factors, which went from 19.7% in 1990 to 24.0% in 2019.

In 1990, 51.2% of the DALYs happened due to NCDs, with 39% attributable to FRs (Figure S1B). In 2019, this percentage had increased to 71.3% and the portion attributable to RFs was 41.1%. The opposite occurred in terms of the DALYs by group, with communicable, maternal, nutritional-neonatal causes. As regards external causes, the burden remained stable during the period, but the portion attributable to RFs went from 18.1% in 1990 to 22.3% in 2019 (Figure S1B).
