# Supplementary material for: Burden of disease attributable to Risk Factors in Brazil: an analysis of national and subnational estimates from the 2019 Global Burden of Disease study
Source: Rev Soc Bras Med Trop. 2022 Jan 28;55(Suppl 1):e0262-2021. doi: 10.1590/0037-8682-0262-2021 (PMC9009437; doi:10.1590/0037-8682-0262-2021)
Supplement: Supplementary file 3 [file 1678-9849-rsbmt-55-s01-e0262-2021-supp3.pdf]

A

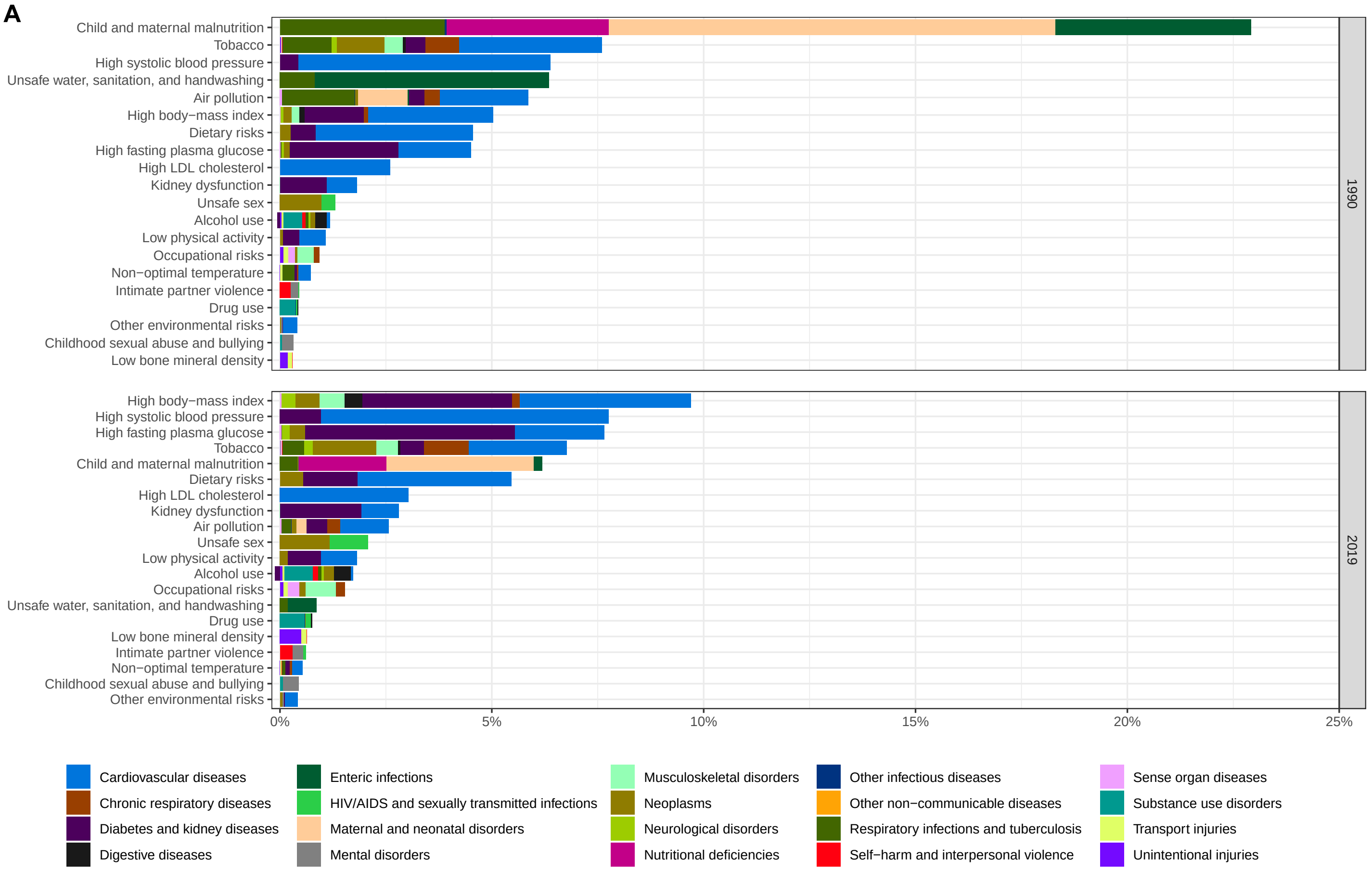

B

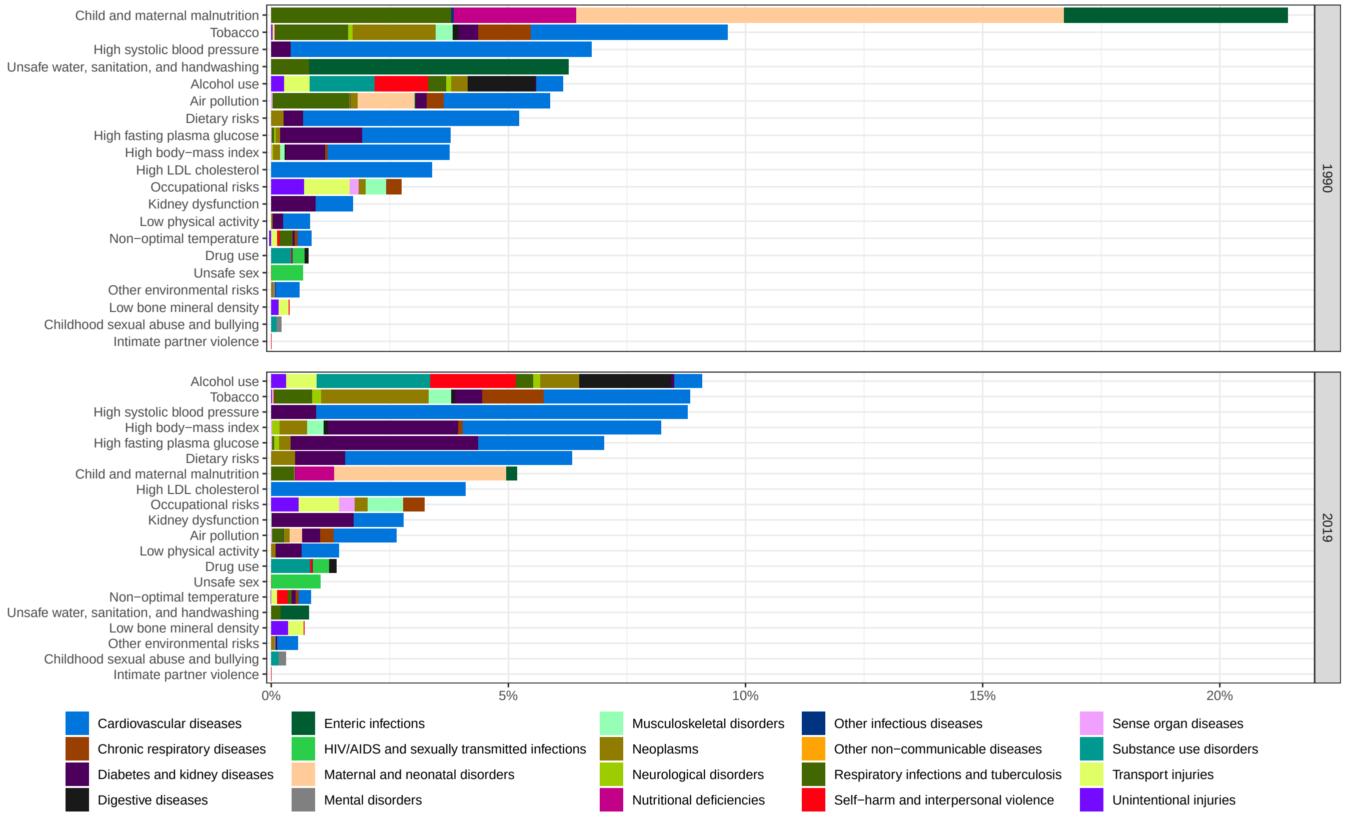

SUPPLEMENTARY FIGURE 2: Percentage of DALYs attributable to RFs (Level 2) for women (A) and for men (B), for all ages, GBD Brazil, 2019.

In terms of the percentage of DALYs in 2019, the main risk factors were the high BMI for women (9.7%), which contributed mostly to DALYs through cardiovascular diseases (4.0%), dia-betes, and kidney disease (3.5%). Among men, the use of alcohol was the main RF, contributing to 9.1% of the total of DALYs, with 2.39% referent to problems related to alcohol consumption and 2.76% to external causes. High systolic blood pressure was the second biggest risk factor for women, which was responsible for 6.78% of the total DALYs in 2019 due to cardiovascular disease, diabetes, and kidney disease. For men, smoking ranked second as a risk factor, which was responsible for 3.09% of the total DALYs due to cardiovascular disease, 2.28% due to neoplasms, and 1.3% due to chronic respiratory disease (Figures S2A and 2B).
