# Supplementary material for: Burden of disease attributable to Risk Factors in Brazil: an analysis of national and subnational estimates from the 2019 Global Burden of Disease study
Source: Rev Soc Bras Med Trop. 2022 Jan 28;55(Suppl 1):e0262-2021. doi: 10.1590/0037-8682-0262-2021 (PMC9009437; doi:10.1590/0037-8682-0262-2021)
Supplement: Supplementary file 4 [file 1678-9849-rsbmt-55-s01-e0262-2021-supp4.pdf]

	Brazil	Acre	Alagoas	Amapá	Amazonas	Bahia	Ceará	Distrito Federal	Espírito Santo	Goiás	Maranhão	Mato Grosso	Mato Grosso do Sul	Minas Gerais	Pará	Paraíba	Paraná	Pernambuco	Piauí	Rio de Janeiro	Rio Grande do Norte	Rio Grande do Sul	Rondônia	Roraima	Santa Catarina	São Paulo	Sergipe	Tocantins
High body−mass index	2405	2344	3183	2323	2282	2596	2261	2332	2574	2336	2793	2439	2493	2081	2373	2584	2374	2919	2262	2743	2437	2252	2590	2666	2167	2280	2654	2687
High systolic blood pressure	2283	2122	3183	1889	1744	2571	2113	1764	2442	2231	2865	1937	2328	1939	2014	2337	2299	2805	2161	2619	2027	2135	2261	2164	2046	2275	2392	2195
Child and maternal malnutrition	2242	3411	2808	3393	2347	3119	2432	1727	2010	2009	2657	1755	2012	2111	2555	1917	1811	2511	2550	1716	1839	1761	2052	3102	1920	1893	2702	2277
Tobacco	2153	2425	2093	1721	1660	1784	1995	1639	1975	2163	1869	1884	2073	1984	1716	1868	2367	2482	1589	2375	1771	2683	2119	1919	2163	2337	1633	1840
High fasting plasma glucose	2024	2113	3287	1976	2043	2429	2044	1833	2010	1912	2931	2011	2017	1572	2213	2511	2036	2785	2172	2298	2299	1751	2250	2539	1751	1705	2611	2313
Dietary risks	1618	1469	2238	1380	1333	1778	1625	1185	1601	1592	2269	1423	1596	1398	1558	1794	1563	2108	1681	1814	1597	1472	1570	1512	1357	1539	1693	1779
Alcohol use	1539	1528	1875	1367	1334	1943	1777	1403	1863	1693	1375	1467	1456	1596	1284	1584	1599	1926	1370	1471	1631	1482	1419	1430	1286	1330	1959	1423
High LDL cholesterol	981	807	1266	800	697	974	968	751	1018	1006	1341	852	1016	827	896	1071	960	1335	930	1167	961	873	966	825	861	964	951	1045
Kidney dysfunction	778	913	1065	862	807	911	705	678	818	814	979	780	753	704	801	852	739	886	709	929	719	696	909	913	628	702	855	883
Air pollution	763	767	1189	637	537	857	719	459	677	706	1284	585	650	736	789	931	647	801	1043	801	735	683	713	667	696	681	770	815
Occupational risks	667	776	660	640	677	701	681	500	664	725	741	696	709	662	702	652	723	678	673	555	645	792	785	676	768	607	648	679
Low physical activity	449	449	640	401	427	467	476	418	448	414	615	407	429	357	455	569	425	589	426	547	452	419	514	495	417	405	503	477
Unsafe sex	404	349	428	484	730	385	372	289	370	339	617	395	379	299	551	351	344	456	388	543	300	583	375	482	410	330	379	390
Drug use	303	294	267	264	457	274	263	301	289	273	291	286	285	300	270	267	278	279	276	342	244	344	277	335	306	331	264	267
Unsafe water, sanitation, and handwashing	281	606	527	299	421	414	388	139	223	262	427	306	278	195	420	327	214	402	314	172	338	187	408	313	214	158	345	336
Non−optimal temperature	198	93	209	239	189	186	402	58	89	127	305	183	151	101	235	221	189	226	385	122	403	241	147	316	270	122	244	294
Low bone mineral density	186	178	185	145	138	187	195	205	249	247	196	220	213	174	167	160	213	169	185	184	149	162	230	222	196	183	213	216
Other environmental risks	140	143	314	127	159	201	184	49	121	150	368	117	138	103	170	206	117	224	221	116	164	121	171	164	89	92	171	198
Childhood sexual abuse and bullying	108	106	115	106	93	90	111	106	110	92	87	100	105	95	83	139	125	118	101	81	106	123	96	105	115	129	116	110
Intimate partner violence	77	79	104	81	73	78	77	71	103	98	80	82	79	65	83	85	70	101	61	69	84	65	87	95	114	67	82	78

SUPPLEMENTARY FIGURE 3: Ranking of RFs according to age-standardized DALY rates for Brazil and its states in 2019, GBD Brazil, 2019.

In terms of DALYs, first place in the ranking was high BMI in half of the states, while maternal and child malnutrition ranked first in Minas Gerais, Ceará, Bahia, Amapá, Amazonas, Acre, Pará, Piauí, Roraima, and Sergipe (Figure S3). Smoking also ranked first in Rio Grande do Sul and São Paulo, while in Maranhão, high systolic blood pressure ranked first. High systolic blood pressure, maternal and child malnutrition, smoking, high fasting plasma glucose, dietary risks, alcohol use, high LDL-cholesterol, kidney dysfunction, and air pollution were among the 10 most important risk factors in most states. Air pollution ranked eighth in Alagoas, Paraíba, Maranhão, and Piauí. It should be highlighted that unsafe sex ranked 9th in Amazonas, while unsafe water, sanitation, and handwashing ranked 12th in Acre; 13th in Alagoas, Amazonas, Bahia, Ceará, and Rondônia; 14th in Maranhão, Pará, and Pernambuco (Figure S3).
