# Supplementary material for: Burden of disease attributable to Risk Factors in Brazil: an analysis of national and subnational estimates from the 2019 Global Burden of Disease study
Source: Rev Soc Bras Med Trop. 2022 Jan 28;55(Suppl 1):e0262-2021. doi: 10.1590/0037-8682-0262-2021 (PMC9009437; doi:10.1590/0037-8682-0262-2021)
Supplement: Supplementary file 5 [file 1678-9849-rsbmt-55-s01-e0262-2021-supp5.pdf]

SUPPLEMENTARY TABLE 1: Socio-Demographic Index, mortality rates attributable to selected RFs (alcohol, high BMI, unsafe water and sanitation, and child and maternal malnutrition), and relative change in mortality between 1990 and 2019, by state.

	SDI	Alcohol use			Child and maternal malnutrition			High body-mass index			Unsafe water, sanitation, and handwashing		
		1990	2019	PC (%)	1990	2019	PC (%)	1990	2019	PC (%)	1990	2019	PC (%)
Acre	0.56	35.7 (27.3;44.9)	32.6 (25.7;40.3)	-8.7 (-31.9;23)	104.5 (89.6;121.8)	35.9 (28.4;44.9)	-65.6 (-74.1;-55.8)	50.9 (23.5;84.4)	75.3 (50.7;102.5)	47.9 (11.7;140.2)	50 (39.2;62.5)	10 (6.9;13.1)	-80 (-85.4;-75.1)
Alagoas	0.52	36 (25.3;48.9)	39.2 (28.2;51.7)	8.7 (-28.8;64.5)	210.9 (178.4;249.4)	29.5 (22.6;37.8)	-86 (-89.9;-81.3)	69.8 (35.1;111.7)	101.1 (69.1;137.2)	44.9 (9;123.8)	90.8 (68.6;121.3)	9.4 (7.1;11.9)	-89.6 (-92.5;-86)
Amapá	0.64	23.6 (16.2;31.6)	26.4 (19.2;33.4)	11.8 (-22.3;59.4)	51.9 (45.9;58.5)	34.9 (29.3;41.6)	-32.7 (-46;-16.7)	54.7 (30.3;83.8)	71.5 (49.3;96.1)	30.6 (6.2;77.4)	20 (16.1;24.1)	5.3 (3.7;7)	-73.5 (-79.6;-67.4)
Amazonas	0.6	35 (25.2;45.4)	27.9 (20.4;36.2)	-20.3 (-46;15.3)	66.1 (56.2;77.8)	23.7 (20.5;27.4)	-64.1 (-70.7;-56.5)	70.6 (40.5;106.3)	71.2 (49;94.7)	0.9 (-17.4;35.7)	36.9 (30.6;43.9)	7.6 (5.5;9.8)	-79.3 (-83.9;-74.5)
Bahia	0.56	30.6 (20.7;42.9)	36.6 (25.9;49.7)	19.7 (-26.1;95.8)	136.1 (115.7;162.4)	32.5 (25.3;40.8)	-76.2 (-82.4;-68.4)	65.3 (33.6;102.3)	81.1 (54.1;113.1)	24.1 (-5.4;86.8)	59.5 (47.4;76.1)	6.4 (4.7;8.4)	-89.3 (-92.5;-85.7)
Ceará	0.56	29.4 (20.9;38.6)	36 (26.1;48.7)	22.5 (-17.7;89.5)	144.7 (121.4;176.7)	24.4 (18.7;30.9)	-83.2 (-87.7;-77.4)	43.8 (20.4;72.5)	72.5 (48.2;101.2)	65.4 (19.9;172.1)	65.2 (49.2;86.6)	8 (5.6;10.6)	-87.7 (-91.6;-82.8)
Distrito Federal	0.78	44.2 (30.1;59)	30.6 (22.5;39)	-30.8 (-52.9;5.2)	46.3 (40.9;52.5)	17.7 (14.9;20.6)	-61.8 (-69;-53.2)	121.9 (75.5;176.5)	80.6 (54.6;107.6)	-33.8 (-45.2;-14.6)	16.9 (12.9;20.7)	3.6 (2.2;5.2)	-78.4 (-84.3;-73)
Espírito Santo	0.66	38.9 (26.8;52.2)	39.5 (29;50.7)	1.5 (-30.8;52)	63.1 (56.1;71.5)	20.7 (16.4;26)	-67.2 (-75.2;-58)	75.8 (40.9;117.6)	83.7 (57.3;112.7)	10.4 (-13;61.5)	18.4 (14.8;22.2)	3.9 (2.5;5.3)	-79.1 (-85.1;-73.5)
Goiás	0.63	40.1 (26.1;54.9)	33.8 (23.8;44.9)	-15.8 (-44.6;29.7)	59.5 (50.9;69.2)	20 (17.1;23.1)	-66.4 (-72.9;-58.2)	76.8 (38.1;126.3)	73.9 (50.3;101.7)	-3.8 (-29.4;49.2)	23.3 (18.2;28.5)	4.4 (2.8;6.2)	-81 (-86.2;-76.3)
Maranhão	0.44	31.3 (19.2;45.7)	28.4 (18.5;40.5)	-9.4 (-45.3;57)	137.6 (114.1;169.3)	26.9 (21.4;33.3)	-80.5 (-85.9;-73.6)	48.2 (18.5;87.4)	89 (56;128.6)	84.8 (25.5;250.7)	49.6 (34.7;67.9)	8.4 (6.1;11.2)	-83 (-88.2;-75.8)
Mato Grosso	0.64	30.8 (19.8;44.7)	29.1 (21.4;38.1)	-5.6 (-40.1;53.6)	57.7 (48.3;68)	17.4 (15;20.1)	-69.8 (-75.7;-62.3)	63.8 (32.6;101.1)	76.8 (52.7;102.4)	20.5 (-9.1;85.8)	24 (18.7;29.4)	4.6 (3.2;6.2)	-80.7 (-85.4;-75.5)
Mato Grosso do Sul	0.64	34.1 (23.4;46.6)	29.5 (21.8;38.8)	-13.5 (-40;28.4)	60.1 (53;68.1)	21 (18;24.3)	-65 (-71.5;-57.1)	83.3 (46.1;128.2)	77 (52;103.9)	-7.6 (-26;24.5)	21.9 (17.7;26.4)	4.9 (3.3;6.4)	-77.7 (-82.3;-73.5)
Minas Gerais	0.64	40.3 (27.6;52.5)	32.7 (24.1;41.3)	-18.7 (-42.3;23.7)	76.4 (66.3;88.4)	21.7 (17.1;27.2)	-71.6 (-78.8;-62.5)	79.5 (41.8;123.4)	62.7 (42.8;84.8)	-21.1 (-36.5;13.9)	20.8 (16.3;25.5)	3.9 (2.4;5.5)	-81.1 (-86.3;-76.5)
Pará	0.57	29.4 (18.8;40.9)	26.1 (18;34.5)	-11.2 (-42.9;39.2)	96.2 (79.9;115)	25.8 (20.1;32.9)	-73.1 (-80.3;-63.9)	65.2 (33.7;103.8)	74 (49.8;100.6)	13.5 (-12.8;70.3)	52.9 (44.2;63.8)	7.7 (5.4;10.1)	-85.5 (-89.5;-81.6)
Paraíba	0.55	28.6 (20.2;38)	32 (22.1;42.1)	11.9 (-27.8;70.8)	110.8 (93.7;134)	19 (14.5;24.2)	-82.9 (-87.8;-76.4)	56.6 (28.1;91.7)	82 (56.8;111.2)	45 (9.4;129.3)	34.2 (25.4;45.2)	5.7 (4;7.6)	-83.3 (-88.5;-77.2)
Paraná	0.66	38.5 (26.8;51.4)	33.7 (24.6;42.7)	-12.5 (-39.2;28.6)	55.9 (49.5;63.4)	17.7 (13.8;22.1)	-68.4 (-76.8;-58.8)	94.2 (52.1;145)	77.1 (50.8;106.2)	-18.2 (-31.9;9.1)	22.3 (18.3;26.3)	4 (2.6;5.5)	-82 (-86.9;-77.2)
Pernambuco	0.57	44.1 (31.1;58.4)	40.1 (28.9;53)	-9 (-37.6;36.1)	155 (130.9;186.2)	27.5 (21.9;33.9)	-82.3 (-86.9;-76.6)	75.4 (38.8;118.8)	95.3 (65.8;128.2)	26.3 (0.7;83.6)	79.6 (64.7;98.5)	7.6 (5.8;9.6)	-90.4 (-92.9;-87.8)
Piaui	0.51	23.7 (15.4;33.8)	25.5 (18.1;34)	7.6 (-31.6;71.2)	113.9 (96.4;135.6)	26.2 (20;33.1)	-77 (-83.3;-69.3)	49.6 (20.4;86.2)	70.1 (47.1;94.8)	41.3 (3.1;159.1)	44.1 (33.9;56.5)	6.1 (4.6;7.9)	-86.1 (-89.8;-81.6)
Rio de Janeiro	0.7	54.5 (39;69.9)	33.5 (24.5;43.4)	-38.5 (-56.3;-9.3)	62 (54.9;70.3)	17.1 (14.9;19.3)	-72.4 (-77.4;-67)	120.7 (72.9;175.6)	87.3 (60.2;117.2)	-27.7 (-39.7;-6.8)	18.1 (13.5;23)	4.4 (2.5;6.4)	-75.5 (-82.8;-68.7)
Rio Grande do Norte	0.58	25 (16.8;36.1)	32.1 (21.7;44.4)	28 (-19.6;102)	105.9 (89.5;127.3)	17.8 (14;22.2)	-83.2 (-87.9;-77.6)	53.2 (25.5;85.9)	75.7 (50.4;104.7)	42.2 (4.4;128.1)	49.3 (39.2;62.5)	5.6 (4;7.4)	-88.6 (-91.7;-84.9)
Rio Grande do Sul	0.68	37.3 (25.1;50.5)	31.8 (22.3;41.3)	-14.8 (-42.5;31.3)	39.3 (35;44.1)	17 (14.7;19.5)	-56.6 (-64.2;-48)	96.3 (56.6;142.5)	73.8 (49.1;101.1)	-23.3 (-34.4;-4.2)	10.7 (7.9;13.5)	3.2 (1.9;4.6)	-69.6 (-76.4;-63.6)
Rondônia	0.61	40.4 (28;54.7)	28.7 (21.3;37.8)	-28.8 (-52.8;12)	73.4 (63.4;85.5)	20.4 (17.3;23.9)	-72.3 (-78.2;-64.9)	92.2 (48.4;144.4)	84.2 (57.6;113.1)	-8.7 (-29.9;33.9)	42.6 (33.8;50.8)	7.2 (5.1;9.5)	-83 (-86.9;-78.6)
Roraima	0.61	40.1 (28;53.5)	29.6 (21.3;37.7)	-26.1 (-49.9;7.7)	80.1 (70;91.5)	33.8 (29.3;38.7)	-57.8 (-65;-48)	84.5 (44.7;131.4)	91 (64.2;119.8)	7.8 (-14.7;58.5)	31.9 (25.7;38.9)	6.9 (4.9;9)	-78.4 (-83;-73.8)
Santa Catarina	0.69	34.3 (21.1;49)	26.7 (18.5;35.7)	-22 (-51.1;30.4)	49.6 (42;58.3)	18.5 (14.1;23.4)	-62.7 (-72.8;-50.5)	93.3 (55.9;139.1)	72.1 (49;96.8)	-22.7 (-35.5;-1.7)	17.1 (13.5;20.8)	3.6 (2.3;4.9)	-79 (-84.3;-73.8)
São Paulo	0.7	47.7 (35.9;59.6)	29.4 (22.6;36.6)	-38.2 (-54.6;-15)	61.2 (54.1;70.3)	16.9 (14.6;19.2)	-72.4 (-77.3;-67.1)	102.9 (60.7;151.2)	72.4 (49.6;97.8)	-29.6 (-41;-9.3)	16.6 (12;21.3)	4 (2.3;5.9)	-75.8 (-82.4;-69.9)
Sergipe	0.58	36.2 (26.3;47.4)	39.6 (28.6;52)	9.2 (-26.6;64.8)	115.7 (98.9;135.7)	27.8 (22.4;33.6)	-76 (-81.8;-68.9)	73.4 (39.6;112.1)	84.4 (57.5;114.3)	14.9 (-11.8;65.8)	58.1 (47.4;71.7)	6.5 (4.6;8.4)	-88.9 (-91.8;-85.6)
Tocantins	0.58	23.6 (15;34.2)	28.6 (19.9;38.4)	21.1 (-25.1;92.8)	96.9 (80.2;117.8)	23.3 (18.8;29.2)	-76 (-82.3;-67.5)	56.9 (24.3;99.6)	89.4 (59.9;122.4)	57.1 (11.6;185)	40 (30.3;53)	5.2 (3.8;6.7)	-87 (-90.9;-82.8)
